# The characteristic of poverty-alleviation behavioral strategies of the rural poor population and their relationship with socio-demographic factors

**DOI:** 10.3389/fpsyg.2024.1450291

**Published:** 2024-12-30

**Authors:** Yan Li, Jin-wei Zhu, Yong-quan Huo

**Affiliations:** ^1^Faculty of Education, Shaanxi Xueqian Normal University, Xi'an, Shaanxi, China; ^2^School of Psychology, Shaanxi Normal University, Xi’an, Shaanxi, China

**Keywords:** rural poor population, poverty-alleviation behavioral strategies, socio-demographic characteristics, factor analysis, hierarchical regression analysis

## Abstract

**Objective:**

The primary objective of this study is to investigate the fundamental patterns and characteristics of poverty-alleviation behavior strategies among rural poor population. It aims to examine the association between the key socio-demographic characteristics of these populations and their poverty-alleviation strategies, thereby identifying the individual and sociocultural factors related to these behaviors.

**Methods:**

This study employs a questionnaire designed to assess poverty-alleviation behavior strategies among rural poor population. Field surveys were conducted in over 80 natural villages across eight provinces and regions in central, northwestern, and southwestern China, involving 1,457 rural poor population. The data collected were analyzed using a questionnaire assessment method to explore the relationship between poverty-alleviation behavior strategies and socio-demographic factors.

**Results:**

Factor analysis indicates that the poverty-alleviation behavior strategies of rural poor population primarily manifest as fatalism, pragmatism, skepticism, and helplessness. Reliability and validity analyses demonstrate that the Poverty-alleviation Behavior Strategies Questionnaire exhibits high internal consistency reliability, composite reliability, convergent validity, and discriminant validity. Hierarchical regression analysis reveals that socio-demographic characteristics such as health status, educational level, family size, main occupation, and income sources have predictive power regarding the formation of pragmatic and fatalistic poverty-alleviation strategies.

**Conclusion:**

Among the four types of poverty-alleviation behavior strategies identified among rural poor population, fatalistic and pragmatic strategies are critically significant for poverty-alleviation efforts and should receive heightened attention from poverty-alleviation workers. While socio-demographic characteristics have limited explanatory power for poverty-alleviation strategies, the sociocultural foundations of pragmatic and fatalistic strategies are relatively more pronounced. Improving the socio-demographic characteristics of rural poor population can, to some extent, facilitate the adoption of pragmatic poverty-alleviation strategies and mitigate the reliance on fatalistic strategies. This conclusion has practical implications for conducting poverty-alleviation work in rural areas.

## 1 Introduction

Poverty constitutes one of the paramount social challenges confronting the global community, exerting profound constraints on the socioeconomic advancement of nations worldwide. It also acts as a catalyst for regional conflicts, terrorism, racial discrimination, and environmental degradation. Consequently, inquiries into the origins and management of poverty have ascended to a position of prominence within the purview of international scholarly discourse. The United Nations has underscored the imperative of “eradicating all forms of poverty globally” as a cornerstone objective within its 2030 Agenda for Sustainable Development, comprising 17 Sustainable Development Goals (SDGs). Both governmental and non-governmental entities across the globe have mobilized substantial resources and efforts toward mitigating poverty. China, being the developing nation with the highest concentration of rural poor population, has accorded heightened significance to the governance of rural poverty. In this regard, China has formulated a distinctive poverty reduction strategies imbued with Chinese characteristics, thereby making a substantive contribution to the global endeavor of poverty-alleviation (Liu et al., 2018).

Historically, poverty was perceived as an economic phenomenon characterized by the inability of individuals or families to meet their basic subsistence requirements through financial income. The classical economist Smith (1776) pioneered the definition of poverty as “the inability to afford the necessities of life.” This seminal definition has exerted a wide-ranging and profound influence; even into the 20th century, economic researchers and international organizations retained the core principles of Smith (1776) conceptualization. For example, Rowntree (1901) defined poverty as “an income insufficient to sustain the minimal physical necessities”; Townsend (1979) described poverty as “the absence of resources needed to participate in activities, customs, and diets generally accepted by society”; while the World Bank defined poverty as “a state of being poor when some people, families, or groups lack the resources to attain what is commonly recognized within that society as a decent standard of living, including food, housing, clothing, and opportunities for participation” (World Bank, 1981). Over time, the notion of poverty evolved into a more concise understanding of lacking the capacity to achieve a minimum standard of living (World Bank, 1990), encompassing “a multidimensional phenomenon that includes lack of opportunity, security, malnutrition, and poor health” (World Bank, 2001). It is evident that since the late 1990s, the comprehension of poverty has broadened from a singular focus on income and expenditure to a multidimensional perspective involving health, housing, education, and social security (Alkire et al., 2017a,b; Pasha, 2017; Alkire and Seth, 2015). Poverty, as a multidimensional construct, denotes the deprivation of human dignity, opportunities, and satisfaction across various dimensions such as food, nutrition, power, education, health, transportation, and income (Alkire and Foster, 2011; Arndt et al., 2016; Chen and Ravallion, 2008; Si et al., 2015). A nuanced understanding of poverty facilitates the objective measurement of poverty phenomena and informs the development and implementation of regional poverty-alleviation policies.

Poverty, characterized by its enduring historical persistence and intricate underlying realities, constitutes a multidisciplinary issue that spans various fields of study. Since the modern British economist Thomas Malthus first drew attention to the issue of poverty, discourse on this topic has been predominantly concentrated within disciplines such as economics, anthropology, sociology, and management studies. In the realm of economics, the emphasis is frequently placed on resource allocation, levels of economic development, and regional growth; anthropology tends to explore ethnic and ecological environments; sociology delves into the social and cultural roots of poverty at a theoretical level; while management studies lean toward policy orientation, researching practical issues related to poverty-alleviation (Luo, 2007). Each of these disciplines has made unique contributions to addressing poverty at the practical level. However, poverty involves not only economic, social, and cultural issues but also encompasses the social psychological dimensions affecting those who experience it.

Studies have demonstrated that the majority of the global poor population resides in rural regions (Oxford Poverty and Human Development Initiative, 2018). In recent decades, research on poverty-alleviation practices in developing countries’ rural areas has garnered increasing attention from scholars (Alkire et al., 2017a,b). In the context of rural poverty-alleviation practices in developing countries, implementing measures such as increasing financial support, developing infrastructure, and improving educational levels plays a crucial role in enhancing the income of the poor population and promoting economic growth in poor regions (Arsani et al., 2020; Cross and Neumark, 2021; Deepika and Sigi, 2014; Mugo and Kilonzo, 2017; Page and Pande, 2018). For example, McLaughlin and colleagues designed poverty-alleviation projects utilizing social psychology framework effects and default effects, advocating for intensified efforts in education, culture, and industrial poverty-alleviation (McLaughlin et al., 2015). Research based on multidimensional poverty theory indicates that the lack of human capital is a primary cause of poverty. Compared to emphasizing income increase, the enhancement of human capital is more likely to effectively improve the living capabilities of the poor population, thereby reducing the incidence of poverty (Norton and Foster, 2001). As Schutz points out in his human capital theory, the fundamental cause of poverty lies not in the shortage of material capital but in the scarcity of human capital and the neglect of investing in human capital (Schultz, 1961). Therefore, developing the human capital of rural poor population is an important pathway for both poverty-alleviation and consolidating the achievements of poverty reduction.

Within the international academic discourse, research on poverty psychology predominantly concentrates on three facets: the attribution of poverty (Feagin, 1972; Wu and Zhang, 2007; Wollie, 2009; Niemela, 2008; Abouchedid and Nasser, 2002; Nasser, 2007; Morçöl, 1997; Pandey et al., 1982; Furnham, 1982), the individual psychological traits of poor populations (Shah et al., 2012; Walker et al., 2013; Gordon et al., 2000; Hobcraft and Kiernan, 2001), and the individual psychological consequences of poverty (Mani et al., 2013; Schilbach et al., 2016; Haushofer and Fehr, 2014). Notably, there is a paucity of studies investigating the behavioral strategies employed by individuals to cope with their state of poverty, thereby creating significant gaps in our understanding of the diversity and complexity of behaviors related to coping with poverty.

Poverty-alleviation strategies employed by rural poor population represent a critical lens through which to examine poverty status and evaluate the efficacy of anti-poverty interventions from a psychological perspective. This paper refers to the behavioral approaches adopted by rural poor population in response to their poverty as “poverty-alleviation behavioral strategies.” These strategies not only serve as direct indicators for observing and assessing how rural poor population manage familial and transactional issues but also function as determinants and predictors of whether the state of poverty can be altered and if the status of being poverty-free is sustainable. Building upon advancements in related research within the international academic community, this study employs a questionnaire assessment method to explore the fundamental patterns and characteristics of poverty-alleviation behavioral strategies among China’s poor population. It investigates the association between key socio-demographic factors of these groups and their poverty-alleviation behavioral strategies to identify the pertinent individual and sociocultural factors influencing these strategies. This research provides robust theoretical and empirical support for advancing our understanding of the psychological mechanisms underlying the poverty-alleviation behaviors of rural poor population and enhancing the scientific rigor of targeted psychological poverty-alleviation services. Given that China’s poor population has historically been concentrated in rural communities within central and western regions, the focus of this study is confined to the rural population in these areas and excludes urban poor residents.

## 2 Methods and procedure

### 2.1 Participants

This study utilized a stratified random sampling approach, targeting eight provinces and regions across central China (Shanxi, Henan, Hubei), northwest China (Shaanxi, Xinjiang, Qinghai), and southwest China (Guizhou and Yunnan). Over 800 villages were randomly selected to include 1,600 individuals aged 20 and above, who were officially identified and registered by local township governments in the “National Poverty Alleviation and Development Information System.” These individuals served as the survey respondents for this study. They completed a self-administered “Poverty Alleviation Behavior Strategies Questionnaire” (see below for details). A total of 1,600 questionnaires were distributed, with 1,512 returned, yielding a response rate of 94.5%. After excluding 88 incomplete or invalid questionnaires, 1,457 valid questionnaires remained for statistical analysis, consisting of 787 males (54%) and 670 females (46%). The 1,457 respondents were randomly assigned to three subsamples: Subsample A (*n* = 486; 274 males and 212 females) was used for exploratory factor analysis of poverty alleviation behavior strategies; Subsample B (*n* = 485; 248 males and 237 females) was used for confirmatory factor analysis of poverty alleviation behavior strategies; Subsample C (*n* = 486; 265 males and 221 females) was used to investigate the predictive impact of socio-demographic factors on poverty alleviation behavior strategies among the rural poor population.

### 2.2 The construction of Poverty-alleviation Behavior Strategies Questionnaire

Firstly, through a comprehensive literature review, theoretical analysis, individual interviews with rural poor population, and consultations with experts and scholars, an initial questionnaire comprising 50 items was preliminarily formulated. Each item in the questionnaire described potential behaviors that individuals might adopt to escape poverty (e.g., “I feel powerless to escape poverty”; “I can respond rationally to family life difficulties”). Respondents were instructed to rate on a 5-point scale (1 = completely disagree; 2 = basically disagree; 3 = somewhat agree; 4 = basically agree; 5 = completely agree) according to their personal experiences and usual behaviors regarding how well each item described their actual situation.

Secondly, employing indicators such as item popularity, item-to-dimension commonality, and dimension loading values for item quality identification, the preliminary survey data (*n* = 154) underwent analysis of item popularity and item discrimination. Consequently, 18 items from the initial Poverty-alleviation Behavior Strategies Questionnaire were retained to form the final “Poverty -alleviation Behavior Strategies Questionnaire.” This survey also included socio-demographic items about the respondents (including age, gender, ethnicity, education level, marital status, family size, health status, main occupation, sources of income, poverty duration, poverty degree, intergenerational poverty, etc.).

## 3 Four-dimensional model of poverty-alleviation behavioral strategies

### 3.1 Items analysis

To evaluate the response tendencies of individuals in poverty toward the poverty alleviation behavior strategy assessment items, and thus obtain information on item quality, the responses of Sample A (*n* = 486) to all items were utilized as a foundation. Based on the formula “*p* = item average score / total item score,” the comprehensibility of all items in the questionnaire was calculated. The results indicated that the *p*-values for all items in the “Poverty-alleviation Behavior Strategy Questionnaire” ranged between 0.50 and 0.70, suggesting that the comprehensibility of all evaluation items is within an acceptable range.

### 3.2 Exploratory factor analysis

(1) Necessity and suitability test of factor analysis. Based on the survey data of sample A, the results of Bartlett’s Test of Sphericity for all evaluation items showed that *χ2* = 2491.910, *df* = 153, *p* < 0.001, indicating that the composition There are shared factors among the various items of the questionnaire, so it is necessary to perform factor analysis on the correlation matrix; the sample fitness test results show that *KMO* = 0.870, which is higher than the expected standard of 0.80, indicating that it is suitable to perform factor analysis on the correlation matrix.

(2) Common factor extraction is based on the basic principles of factor analysis, using *α* factor decomposition and Promax oblique rotation method for factor extraction. The results show four common factors with characteristic root *λ* > 1. Referring to the results of the steep order test, the first four common factors can explain 54.085% of the total variation of the questionnaire. Moreover, the commonality of the factors corresponding to each item that constitutes the Poverty-alleviation Behavior Strategies Questionnaire is above 0.50, which meets the basic statistics requirements.

(3) Common factor rotation and factor naming. In order to make the loading form on each dimension the simplest, the variance of the absolute values of all loadings on each dimension reaches the relative maximum, thereby generating a central dimension (Xie, 1989) using Promax. The oblique rotation method rotates the four extracted dimensions. The results (see Table 1) show that after Promax oblique rotation, the item loading coefficient has different loading values on the factors. Based on this, this item is classified as having the highest loading. The five items of dimension I reflect the negative attitude of rural poor population toward their behavior in getting rid of poverty, which is named “fatalism,” can explain 29.14% of the total variation; the five items of dimension II reflect the ability and confidence of rural poor population to get out of poverty. Family poverty and optimism about the future are named “Pragmatism,” which can explain 12.30% of the total variation; the five items in Dimension III reflect the poor people’s skepticism about the effectiveness of poverty-alleviation, named “Skepticism” can explain 7.08% of the total variation; the three items of dimension IV reflect the fact that rural poor population feel that they are not supported by society and others in the practice of poverty-alleviation, and are named “helplessness,” which can explain 5.56% of the total variation. Total variation. Among the four public dimensions, the two dimensions of “fatalism” and “pragmatism” have the highest explanation rate of the total variation, with their joint explanatory power reaching 41.45%.

**Table 1:** Items’ factor loading of Poverty-alleviation Behavior Strategies Questionnaire.

Item number	Item	Factor I	Factor II	Factor III	Factor IV
A09	I do not think I can get rich no matter how hard I try.	0.696		0.415	0.412
A10	I feel it is difficult for me to integrate into the present society.	0.641		0.456	0.476
A15	I feel that my life is meaningless.	0.636		0.406	0.427
A08	I feel like a worthless person.	0.606			
A22	I think the rich and the poor are predestined.	0.566		0.429	
A01	I can concentrate on dealing with my family life.		0.692		
A02	I have the ability to make decisions about family life.		0.656		
A03	I can face the real problems of my family.		0.629		
A04	I am confident that I can get rid of family poverty.		0.561		
A05	I can deal with the difficulties of family life rationally.		0.525		
A24	I have no clear goals and plans in my life.	0.460		0.633	
A25	I think there are more negative things than positive things in life.	0.424		0.631	
A36	I find it difficult to adapt myself to the development of society.			0.563	0.454
A35	I feel incapable of getting rid of poverty.			0.562	
A29	I have no expectations for the future.	0.433		0.473	
A11	I feel helpless in my family life.	0.578			0.617
A12	I cannot control my emotions in the face of family troubles.	0.457			0.612
A14	I feel inferior to others in all aspects.	0.508			0.606

Table 1 omits the load value with absolute value < 0.40.

### 3.3 Confirmatory factor analysis of the strategic dimension of poverty-alleviation behavior

In order to further verify the cross-sample universality of the four-dimensional model of poverty-alleviation behavioral strategies and examine the model’s construct validity, confirmatory factor analysis technology was used to test the fit of the four-dimensional model to the sample B survey data, the specific method is to use AMOS 21.0 software and the maximum likelihood method to estimate the model’s goodness of fit to the data. The model test’s main fitting parameters and indices (see Table 2) show all fitting indices.

**Table 2:** Results of confirmatory factor analysis of Poverty-alleviation Behavior Strategies Questionnaire (*n* = 485).

Index	*χχ2/df*	*RMSEA*	*SRMR*	*CFI*	*TLI*
Theoretical value	<3	<0.1	<0.08	>0.9	>0.9
Actual value	2.635	0.058	0.049	0.904	0.886
Inspection conclusion	Ideal	Ideal	Ideal	Ideal	Reasonable

**Figure 1:**
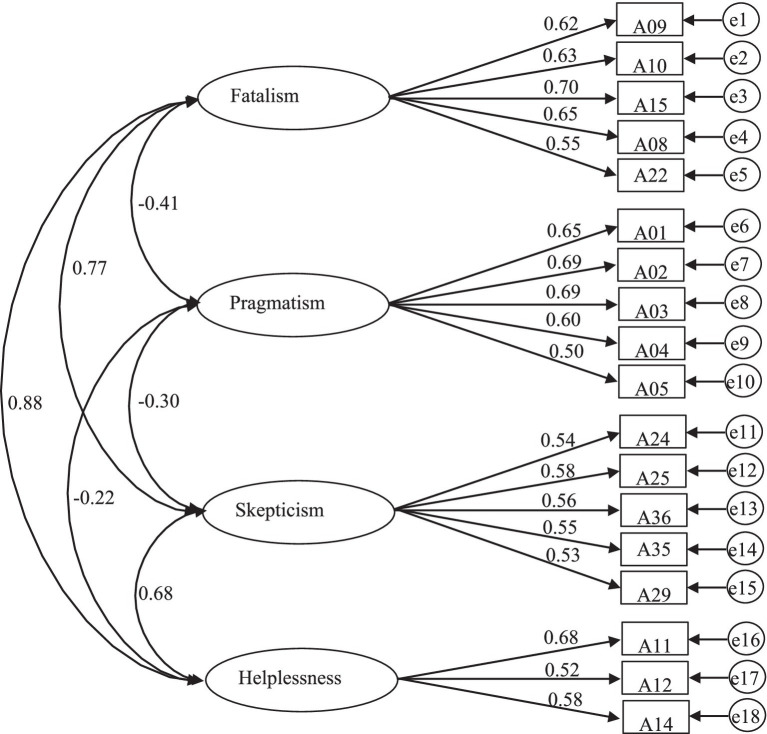
Confirmatory factor analysis model diagram of poverty-alleviation behavioral strategies.

All are within the range of theoretical values, indicating that the adaptation index of the parameter estimation results is good. The four-dimensional model obtained based on sample A is consistent with the survey data of sample B, and the overall model has a good fit. The standardized factor loadings of each item were calculated separately. The loading values of all items were statistically significant (*ps* < 0.001), and the loading values were all above 0.50 (see Figure 1), indicating that each item can effectively reflect the desired measured model constructs (Liden and Maslyn, 1998).

### 3.4 Reliability and validity of the questionnaire on poverty-alleviation behavioral strategies

#### 3.4.1 Reliability analysis

Based on the survey data of Sample A, the Cronbach’s alpha coefficient of each component dimension of the questionnaire was calculated using a formula $\alpha =\frac{K}{K-1}\times \left(1-\sum \frac{S_{i}^{2}}{S_{\mathrm{total}}^{2}}\right)$ to estimate the internal consistency reliability of the questionnaire. The test results (see Table 3) show that theαcoefficients of the four factors are all above 0.70, indicating that the internal consistency reliability of the four dimensions that make up the questionnaire is good.

**Table 3:** Reliability coefficient (α) of each dimension of Poverty-alleviation Behavior Strategies Questionnaire.

Dimension order	Dimension name	Number of entry	*α*
Dimension I	Fatalism	5	0.766
Dimension II	Pragmatism	5	0.745
Dimension III	Skepticism	5	0.720
Dimension IV	Helplessness	3	0.702

#### 3.4.2 Validity analysis

##### 3.4.2.1 Item of distinction

Taking the item distinction as an indicator and based on the survey data of Sample A, the correlation coefficient between the item evaluation score and the total score of the dimension was calculated to identify the effectiveness of each item on the total score of the dimension (item validity). The analysis results (Table 4) show that the correlation between the scores of all 18 items that constitute the poverty-alleviation behavioral strategies questionnaire and the total score of the corresponding dimension has reached the significance level (*p* < 0.001), and all have reached high correlation (*r_s_* > 0.63). It shows that all items have sufficient discrimination for the dimensions to be assessed, thus ensuring the validity of the assessment of each dimension.

**Table 4:** Discriminatory coefficient (R) of each evaluation item in the Poverty-alleviation Behavior Strategies Questionnaire.

Item	*r*	Item	*r*	Item	*r*
A1	0.756^★^	A7	0.746^★^	A13	0.703^★^
A2	0.725^★^	A8	0.721^★^	A14	0.686^★^
A3	0.708^★^	A9	0.798^★^	A15	0.719^★^
A4	0.673^★^	A10	0.796^★^	A16	0.632^★^
A5	0.654^★^	A11	0.781^★^	A17	0.712^★^
A6	0.707^★^	A12	0.719^★^	A18	0.686^★^

△*p* < 0.05; ☆*p* < 0.01; ★*p* < 0.001 (the same below).

##### 3.4.2.2 Composite reliability and convergent validity

In order to evaluate the combined reliability and convergent validity of the model, based on the survey data of sample A, the adaptation of each dimension of the poverty-alleviation behavioral strategies was further tested by calculating the combined reliability (CR) and average variance extracted (AVE) of the latent variables. Sex. Among them, the combined reliability above 0.6 indicates a high degree of internal correlation between the measured indicators. The calculation formula is: ρv = (*Σλ*)^2^/[(Σλ)^2^ + Σ(1–R^2^)]; average variance extraction The critical value of the quantity is 0.5. Exceeding this value indicates that the observed variable can effectively reflect the latent variable it represents. The calculation formula is AVE = (Σλ^2^)/[(Σλ^2^) + Σ(1–R^2^)] (where: λ is Standardized factor loadings of indicators, R^2^ is the square of the standardized coefficient). The software designed by (Wu, 2010) and Excel were used to calculate each dimension’s CR and AVE values. The results (see Table 5) show that the values of each indicator meet the standards, indicating that this structural model has certain convergent validity.

**Table 5:** CR analysis and AVE analysis results of the structural model of poverty-alleviation behavior strategies.

Dimension order	Dimension name	*CR*	*AVE*
Dimension I	Fatalism	0.767	0.398
Dimension II	Pragmatism	0.766	0.398
Dimension III	Skepticism	0.685	0.304
Dimension IV	Helplessness	0.619	0.354

##### 3.4.2.3 Discriminant validity

In order to test the discriminant reliability of the questionnaire, the correlation coefficient between each dimension was used as an indicator to calculate the correlation between the assessment scores of each dimension in the A sample data and the B sample data. The results (see Table 6) show that, first of all, despite all The correlations between dimensions are all significant, but the correlation coefficients between each dimension are all below medium, indicating that although there is a specific connection between these dimensions, they are relatively independent of each other and have sufficient discriminant validity; secondly, for getting rid of poverty There is a negative correlation between the positive “pragmatism” strategies and the remaining three strategies that have a negative significance for getting rid of poverty, and the correlation coefficients between the three last strategies that have a negative significance for getting rid of poverty are all positive, indicating that positive There is antagonism between behavioral strategies and negative behavioral strategies, while there is compatibility between negative behavioral strategies; finally, in the same dimension, the correlation coefficients of the two samples A and B are highly consistent, indicating that the correlation between each dimension is in two There is stability in the sample.

**Table 6:** Correlation coefficient among dimensions of Poverty-alleviation Behavior Strategies Questionnaire (n_A_ = 486/n_B_ = 485).

Dimension name	Fatalism	Pragmatism	Skepticism	Helplessness
Fatalism	1/1			
Pragmatism	−0.296^★^/−0.328^★^	1/1		
Skepticism	0.580^★^/0.566^★^	−0.262^★^/−0.231^★^	1/1	
Helplessness	0.612^★^/0.596^★^	−0.144^☆^/−0.165^★^	0.486^★^/0.451^★^	1/1

△*p* < 0.05; ☆*p* < 0.01; ★*p* < 0.001. The value after “/” is the result of calculation based on group B data.

## 4 The prediction of socio-demographic characteristics

In order to examine the predictive effect of the socio-demographic characteristics of the poor on their poverty-alleviation behavioral strategies, the socio-demographic characteristics of the poor were constructed based on the results of correlation analysis of sample C (*n* = 486) with each dimension of the poverty-alleviation behavioral strategies as units. The hierarchical regression model of each dimension was used to explore the individual and socio-cultural basis of poverty-alleviation behavioral strategies by identifying the gain validity of these socio-demographic characteristics on each dimension of poverty-alleviation behavioral strategies.

### 4.1 Correlation between socio-demographic characteristics and poverty-alleviation behavior strategies

First, the data from sample C (*n* = 486) were used to calculate the Eta series correlation method between each dimension of poverty-alleviation behavioral strategies and the age, education level, health status, family size, poverty duration, poverty degree, main occupation, and source of income of the subjects, intergenerational poverty and other socio-demographic characteristics, and use Eta correlation analysis technology $F=\frac{E^{2}/\left(k-1\right)}{\left(1-E^{2}\right)/\left(n-k\right)}$ (In the formula: E = Eta correlation coefficient; k = number of variable categories; n = sample size) (Wen and Xz, 2001) tested the significance of the Eta coefficient, and the results (see Table 7) show: (1) Fatalistic strategies are significantly correlated with health status, poverty duration, main occupation, source of income, and intergenerational poverty; (2) Pragmatic strategies are significantly correlated with age, education level, poverty degree, main occupation, and source of income; (3) The Skeptical strategies is significantly related to health status and poverty duration; (4) The helplessness strategies is significantly related to the poverty duration. Generally speaking, each dimension that constitutes behavioral strategies for poverty-alleviation has a specific correlation with the characteristics of the poor, their families, and society. However, this correlation shows different patterns, indicating that the socio-demographic characteristics of the poor impact behavioral strategies for poverty-alleviation. Each dimension may have different predictive effects. Accordingly, hierarchical regression analysis techniques were used to construct a cumulative model to examine the predictive role of socio-demographic characteristics on behavioral strategies for poverty-alleviation.

**Table 7:** The correlation between socio-demographic characteristics and dimensions of poverty-alleviation behavior strategies.

Social demographic characteristics		Fatalism		Pragmatism		Skepticism		Helplessness	
		*Eta*	*F*	*Eta*	*F*	*Eta*	*F*	*Eta*	*F*
Individual characteristics	Age (*df* = 3,482)	0.113	2.078	0.163	4.399^☆^	0.118	2.277	0.124	2.503
	education level (*df* = 3,482)	0.093	1.413	0.132	2.865^△^	0.099	1.592	0.073	0.853
	Health status (df = 2,483)	0.162	6.503^☆^	0.090	1.951	0.151	5.630^☆^	0.077	1.451
Family characteristics	Family size (*df* = 2,483)	0.051	0.640	0.094	2.164	0.041	0.416	0.065	1.020
	Poverty duration (*df* = 3,482)	0.182	5.489^★^	0.106	1.833	0.129	2.725^△^	0.150	3.711^△^
	Poverty degree (*df* = 2,483)	0.075	1.348	0.119	3.457^△^	0.084	1.707	0.033	0.267
Social characteristics	Main occupation (*df* = 3,482)	0.140	3.191^△^	0.162	4.345^☆^	0.100	1.621	0.119	2.305
	Income sources (*df* = 2,483)	0.136	4.534^△^	0.210	11.158^★^	0.083	1.664	0.063	0.969
	Intergenerational poverty (*df* = 2,483)	0.114	3.155^△^	0.068	1.129	0.072	1.248	0.070	1.174

△*p* < 0.05; ☆*p* < 0.01; ★*p* < 0.001.

### 4.2 The predictive effect of social demographic characteristics on fatalistic strategies

(1) The gain validity of the individual characteristics of people with low incomes on the fatalistic strategies. Table 8 presents the results of the hierarchical regression analysis of the gain validity of the individual characteristics of people with low incomes (age, health status, education level) on the fatalistic strategies. Among them, in the first step of regression analysis, the regression coefficient of the predictor “age” is not significant (*t* = 0.332, *p* = 0.740), and the determination coefficient of the model is also not significant (*F* = 0.111, *p* = 0.740). This factor cannot effectively explain the dependent variable; in the second step of regression analysis, the regression coefficient of the new predictor “health status” is significant (*t* = 3.538, *p* < 0.001). The decision system of the new model is the number is significant (*F* = 6.316, *p* = 0.002), and the new factor’s explanation rate for the dependent variable is 2.5%; in the third step of regression analysis, the regression coefficient of the new predictor “education level” is not significant (*t* = −1.224, *p* = 0.222). However, the coefficient of determination of the new model is significant (*F* = 4.714, *p* = 0.003), and the additional explanation rate of the new factor on the dependent variable is 0.4%. In models 1–3, as the number of predictors increases, the R^2^ value increases, indicating that the explanatory power of the predictor variables for the dependent variable increases. The two factors of poor people’s health status and education level can effectively explain the fatalistic strategies. The regression equation model is Y = 2.582 + 0.162×X_1_ − 0.051×X_2_ (where X_1_ = health status, X_2_ = education level), and its overall explanation is that the rate is 2.9%.

**Table 8:** The results of hierarchical regression analysis of the individual characteristics of the poor against fatalistic strategies.

Model		Non-standardized coefficient		Standard coefficient Beta	*t*	*F*	*R* ^2^
		*B*	*SE*				
	Constant	2.584	0.107		24.149^★^	0.111	0.000
	Age	0.013	0.038	0.015	0.332		
	Constant	2.390	0.119		20.065^★^	6.316^☆^	0.025
	Age	−0.037	0.040	−0.044	−0.920		
	Health status	0.172	0.048	0.170	3.538^★^		
	Constant	2.582	0.197		13.110^★^	4.714^☆^	0.029
	Age	−0.052	0.042	−0.063	−1.245		
	Health status	0.162	0.049	0.160	3.291^★^		
	Education level	−0.051	0.042	−0.060	−1.224		

△*p* < 0.05; ☆*p* < 0.01; ★*p* < 0.001 (same later).

(2) The gain validity of the family characteristics of low-income people on the fatalistic strategies. Table 9 presents the results of the hierarchical regression analysis of the gain validity of the family characteristics of the poor (family size, poverty duration, poverty degree) on the fatalistic strategies. Among them, in the first step of regression analysis, the regression coefficient of the predictor “family size” is not significant (*t* = −1.127, *p* = 0.260), and the determination coefficient of the model is also not significant (*F* = 1.270, *p* = 0.260), this factor cannot effectively explain the dependent variable; in the second step of regression analysis, the regression coefficient of the new predictor “years in poverty” is not significant (*t* = −0.029, *p* = 0.977). The new model the coefficient of determination of is also not significant (*F* = 0.634, *p* = 0.531). This factor cannot effectively explain the dependent variable; in the third step of regression analysis, the new predictor “poverty degree” regression coefficient is not significant (*t* = 1.639, *p* = 0.102), and the coefficient of determination of the new model is insignificant (*F* = 1.320, *p* = 0.267). This factor cannot effectively explain the dependent variable. It can be seen that the family characteristics of the poor cannot effectively predict their fatalistic poverty-alleviation strategies.

**Table 9:** The hierarchical regression analysis results of family characteristics of the poor on fatalistic strategies.

Model		Non-standardized coefficient		Standard coefficient Beta	*t*	*F*	*R* ^2^	Adjusted R^2^
		*B*	*SE*					
1	Constant	2.770	0.141		19.630***	1.270	0.003	0.001
	Family size	−0.067	0.059	−0.051	−1.127			
2	Constant	2.772	0.157		17.627***	0.634	0.003	−0.002
	Family size	−0.067	0.060	−0.051	−1.116			
	Poverty duration	−0.001	0.035	−0.001	−0.029			
3	Constant	2.572	0.199		12.950***	1.320	0.008	0.002
	Family size	−0.072	0.060	−0.055	−1.209			
	Poverty duration	0.008	0.036	0.011	0.235			
	Poverty degree	0.088	0.053	0.075	1.639			

***Indicates significance at the 0.001 level.

(3) The social characteristics of people with low incomes affect the gain validity of fatalistic strategies. Table 10 presents the results of the hierarchical regression analysis of the social characteristics of the poor (intergenerational poverty, main occupation, source of income) on the gain validity of fatalism. Among them, in the first step of regression analysis, the regression coefficient of the predictor “intergenerational poverty” is not significant (*t* = 1.611, *p* = 0.108), and the determination coefficient of the model is also not significant (*F* = 2.594, *p* = 0.108), this factor cannot effectively explain the dependent variable; in the second step of regression analysis, the regression coefficient of the new predictor “main occupation” is not significant (*t* = −0.663, *p* = 0.507), and the decision of the model The coefficient is also not significant (*F* = 1.516, *p* = 0.221), and the new factor cannot effectively explain the dependent variable; in the third step of regression analysis, the regression coefficient of the new predictor “source of income” is significant (*t* = −2.102, *p* = 0.036). However, the model’s coefficient of determination is not significant (*F* = 2.491, *p* = 0.060), and the new factor’s effective explanation rate for the dependent variable is 1.5%. It can be seen that only the income source factor can effectively explain the fatalistic strategies, with an explanation rate of 1.5%, and its regression equation model is: Y = 2.621–0.093X (X = income source).

**Table 10:** The results of hierarchical regression analysis of the social characteristics of the poor against fatalistic strategies.

Model		Non-standardized coefficient		Standard coefficient Beta	*t*	*F*	*R* ^2^	Adjusted R^2^
		*B*	*SE*					
1	Constant	2.446	0.113		21.726***	2.594	0.005	0.003
	Intergenerational poverty	0.080	0.050	0.073	1.611			
2	Constant	2.497	0.136		18.352***	1.516	0.006	0.002
	Intergenerational poverty	0.079	0.050	0.072	1.584			
	Main occupation	−0.023	0.035	−0.030	−0.663			
3	Constant	2.621	0.148		17.718***	2.491	0.015	0.009
	Intergenerational poverty	0.080	0.049	0.073	1.615			
	Main occupation	0.005	0.037	0.006	0.125			
	Income sources	−0.093	0.044	−0.102	−2.102**			

***Indicates significance at the 0.001 level; **Indicates significance at the 0.01 level.

Based on the results of the above analysis, it can be concluded that the fatalistic poverty-alleviation strategies is mainly related to the poor individual’s health status, education level, and income source. These three factors can jointly explain 4.4% of the total variation of the fatalistic strategies. Those poor people with poor physical health, low education, and who rely on traditional farming for family income are more likely to view their poverty status from a fatalistic perspective. They are more likely to form fatalistic poverty-alleviation strategies.

### 4.3 The predictive effect of socio-demographic characteristics on pragmatism strategies

(1) The gain validity of the individual characteristics of people with low incomes on the pragmatic strategies. Table 11 presents the results of the hierarchical regression analysis of the gain validity of the pragmatic strategies on the individual characteristics of people with low incomes (age, health status, education level). Among them, in the first step of regression analysis, the regression coefficient of the predictor “age” is significant (*t* = 2.911, *p* = 0.004).

**Table 11:** The results of hierarchical regression analysis of individual characteristics of the poor against pragmatic strategies.

Model		Non-standardized coefficient		Standard coefficient Beta	*t*	*F*	*R* ^2^	Adjusted R^2^
		*B*	*SE*					
1	Constant	3.071	0.098		31.187***	8.472*	0.017	0.015
	Age	0.101	0.035	0.131	2.911*			
2	Constant	3.226	0.110		29.340***	9.017***	0.036	0.032
	Age	0.140	0.037	0.182	3.822***			
	Health status	−0.137	0.045	−0.146	−3.068*			
3	Constant	2.808	0.181		15.557***	8.912***	0.053	0.047
	Age	0.173	0.038	0.225	4.545***			
	Health status	−0.116	0.045	−0.123	−2.574*			
	Education level	0.111	0.038	0.140	2.902*			

***Indicates significance at the 0.001 level; *Indicates significance at the 0.05 level.

The coefficient of determination of the model is also significant (*F* = 8.472, *p* = 0.004), and the effective explanation rate of this factor for the dependent variable is 1.7%; in the second step of regression analysis, the regression system of the new predictor “health status” is added. The number is significant (*t* = −3.068, *p* = 0.002), and the coefficient of determination of the new model is also significant (*F* = 9.017, *p* < 0.001). The additional explanation rate of the new factor for the dependent variable is 1.9% in the three-step regression analysis, the regression coefficient of the new predictor “education level” is significant (*t* = 2.902, *p* = 0.004), and the determination coefficient of the new model is also significant (*F* = 8.912, *p* < 0.001). The additional explanation rate of the new factors for the dependent variable is 1.7%. In models 1 to 3, as the number of predictors increases, the R^2^ value increases, indicating that the explanatory power of the predictor variables for the dependent variable increases. The three factors of age, health status, and educational level of poor individuals can effectively explain the pragmatic poverty-alleviation strategies. The regression equation model is: Y = 2.808 + 0.173 × X_1_ − 0.116 × X_2_ + 0.111 × X_3_ (where: X_1_ = age, X_2_ = health status, X_3_ = education level), its overall explanation rate is 5.3%.

(2) The gain validity of the low-income family characteristics on the pragmatic strategies. Table 12 presents the results of the hierarchical regression analysis of the low-income family characteristics (family size, poverty duration, poverty degree) on the gain validity of the pragmatic strategies. Among them, in the first step of regression analysis, the regression coefficient of the predictor “family size” is significant (*t* = 2.033, *p* = 0.043), and the determination coefficient of the model is also significant (*F* = 4.134, *p* = 0.043). The effective explanation rate of this factor for the dependent variable is 0.8%; in the second step of regression analysis, the regression coefficient of the new predictor “poverty duration” is not significant (*t* = −0.890, *p* = 0.374), and the new model’s coefficient of determination is also not significant (*F* = 2.462, *p* = 0.086), and the new factor cannot effectively explain the dependent variable; in the third step of regression analysis, the regression coefficient of the new predictor “poverty degree” is significant (*t* = −2.541, *p* = 0.011). The coefficient of determination of the new model is also significant (*F* = 3.812, *p* = 0.010). The additional explanation rate of the new factor on the dependent variable is 1.5%. The poor’s family size and poverty degree factors can effectively explain the pragmatic poverty-alleviation strategies. The regression equation model is Y = 3.424 + 0.125×X_1_ − 0.125×X_2_ (where X_1_ = family size, X_2_ = poverty degree), and its overall explanation is that the rate is 2.3%.

**Table 12:** The hierarchical regression analysis results of family characteristics of the poor against pragmatic strategies.

Model		Non-standardized coefficient		Standard coefficient Beta	*t*	*F*	*R* ^2^	Adjusted R^2^
		*B*	*SE*					
1	Constant	3.083	0.131		23.605***	4.134**	0.008	0.006
	Family size	0.112	0.055	0.092	2.033**			
2	Constant	3.139	0.145		21.589***	2.462	0.010	0.006
	Family size	0.117	0.055	0.096	2.118**			
	Poverty duration	−0.029	0.033	−0.041	−0.890			
3	Constant	3.424	0.183		18.714***	3.812*	0.023	0.017
	Family size	0.125	0.055	0.103	2.270**			
	Poverty duration	−0.043	0.033	−0.059	−1.291			
	Poverty degree	−0.125	0.049	−0.116	−2.541**			

***Indicates significance at the 0.001 level; **Indicates significance at the 0.01 level; *Indicates significance at the 0.05 level.

(3) The gain validity of the social characteristics of the poor on the pragmatic strategies. Table 13 presents the results of the hierarchical regression analysis of the gain validity of the pragmatic strategies on the social characteristics of the poor (intergenerational poverty, main occupation, source of income). Among them, in the first step of regression analysis, the regression coefficient of the predictor “intergenerational poverty” is not significant (*t* = −1.397, *p* = 0.163), and the determination coefficient of the model is also not significant (*F* = 1.950, *p* = 0.163), this factor cannot effectively explain the dependent variable; in the second step of regression analysis, the regression coefficient of the new predictor “main occupation” is significant (*t* = −2.125, *p* = 0.034), and the new The coefficient of determination of the model is also significant (*F* = 3.241, *p* = 0.040), and the effective explanation rate of the new factor on the dependent variable is 1.3%; in the third step of regression analysis, the regression coefficient of the new predictor “source of income” is It is not significant (*t* = 1.571, *p* = 0.117), but the coefficient of determination of the new model is significant (*F* = 2.990, *p* = 0.031), and the additional effective explanation rate of the new factor for the dependent variable is 0.5%. It can be seen that the main occupation and income source factors of the poor can effectively explain the pragmatic poverty-alleviation strategies. The regression equation model is: Y = 3.539–0.088×X_1_ + 0.064×X_2_ (where, X_1_ = main occupation, X_2_ = income source), and its overall explanation the rate is 1.8%.

**Table 13:** The results of hierarchical regression analysis of social characteristics of the poor against pragmatic strategies.

Model		Non-standardized coefficient		Standard coefficient Beta	*t*	*F*	*R* ^2^	Adjusted R^2^
		*B*	*SE*					
1	Constant	3.476	0.105		33.237***	1.950	0.004	0.002
	Intergenerational poverty	−0.064	0.046	−0.063	−1.397			
2	Constant	3.626	0.126		28.814***	3.241**	0.013	0.009
	Intergenerational poverty	−0.068	0.046	−0.067	−1.480			
	Main occupation	−0.069	0.032	−0.096	−2.125**			
3	Constant	3.539	0.137		25.815***	2.990**	0.018	0.012
	Intergenerational poverty	−0.069	0.046	−0.068	−1.501			
	Main occupation	−0.088	0.035	−0.123	−2.548**			
	Income sources	0.064	0.041	0.076	1.571			

***Indicates significance at the 0.001 level; **Indicates significance at the 0.01 level.

Based on the above analysis results, it can be believed that the formation of pragmatic poverty-alleviation strategies is not only related to the age, health status, education level, and other individual characteristics of the poor (these three factors can explain 5.3% of the total variation of pragmatic poverty-alleviation strategies), but also to The family size and poverty degree of the poor (these two factors can explain 2.3% of the total variation in the pragmatic poverty-alleviation strategies), as well as social characteristics such as the main occupation and income source of the poor (these two factors can explain the pragmatic poverty-alleviation strategies 1.8% of the total variation), with a joint explanation rate of 9.4%. Poor older people, in better health, more educated, have smaller families, have lower levels of poverty, and rely on processing and urban employment for economic income are more likely to view their poverty from a pragmatic perspective because it is easier to form a pragmatic poverty-alleviation strategies.

### 4.4 Prediction of socio-demographic characteristics on skeptic strategies

(1) The gain validity of the individual characteristics of people with low incomes on the Skeptical strategies. Table 14 presents the results of the hierarchical regression analysis of the gain validity of the Skeptical strategies on the individual characteristics of people with low incomes (age, health status, education level). Among them, in the first step of regression analysis, the regression coefficient of the predictor “age” is not significant (*t* = 0.694, *p* = 0.488), and the determination coefficient of the model is also not significant (*F* = 0.481, *p* = 0.488). This factor cannot effectively explain the dependent variable; in the second step of regression analysis, the regression coefficient of the new predictor “health status” is significant (*t* = 3.060, *p* = 0.002), and the coefficient of determination of the new model It is also significant (*F* = 4.926, *p* = 0.008), and the new factor’s explanation rate for the dependent variable is 2.0%; in the third step of regression analysis, the regression coefficient of the new predictor “education level” is not significant (*t* = −1.296, *p* = 0.196). However, the coefficient of determination of the new model is significant (*F* = 3.849, *p* < 0.001), and the additional explanation rate of the new factor on the dependent variable is 0.3%. In models 1 to 3, as the number of predictors increases, the R^2^ value increases, indicating that the explanatory power of the predictor variables for the dependent variable increases. The two individual factors of the poor’s health status and education level can effectively explain their skeptical poverty-alleviation strategies. The regression equation model is: Y = 2.676 + 0.122×X_1_ − 0.048×X_2_ (where: X_1_ health status, X_2_ = education level); its overall explanation rate is 2.3%.

**Table 14:** The results of hierarchical regression analysis of individual characteristics of the poor to skeptical strategies.

Model		Non-standardized coefficient		Standard coefficient Beta	*t*	*F*	*R* ^2^	Adjusted R^2^
		*B*	*SE*					
1	Constant	2.644	0.095		27.956***	0.481	0.001	−0.001
	Age	0.023	0.033	0.032	0.694			
2	Constant	2.495	0.106		23.625***	4.926*	0.020	0.016
	Age	−0.015	0.035	−0.020	−0.412			
	Health status	0.132	0.043	0.147	3.060*			
3	Constant	2.676	0.175		15.322***	3.849*	0.023	0.017
	Age	−0.029	0.037	−0.039	−0.783			
	Health status	0.122	0.044	0.137	2.808*			
	Education level	−0.048	0.037	−0.064	−1.296			

***Indicates significance at the 0.001 level; *Indicates significance at the 0.05 level.

(2) The gain validity of the family characteristics of people with low incomes is based on the skeptical strategies. Table 15 presents the results of the hierarchical regression analysis of the gain validity of the Skeptical strategies on the family characteristics of the poor (family size, poverty duration, and poverty degree). Among them, in the first step of regression analysis, the regression coefficient of the predictor “family size” is not significant (*t* = −0.662, *p* = 0.509), and the determination coefficient of the model is also not significant (*F* = 0.438, *p* = 0.509), this factor cannot effectively explain the dependent variable; in the second step of regression analysis, the regression coefficient of the new predictor “poverty duration” is not significant (*t* = −0.817, *p* = 0.414), and the new model The coefficient of determination of is also not significant (*F* = 0.552, *p* = 0.576), and the new factor cannot effectively explain the dependent variable; in the third step of regression analysis, the regression coefficient of the new predictor “poverty degree” is not significant (*t* = 1.488, *p* = 0.137), and the coefficient of determination of the new model is not significant (*F* = 1.107, *p* = 0.346). The new factor cannot effectively explain the dependent variable. It can be seen that the family characteristics of the poor cannot effectively explain the skeptical strategies of poverty-alleviation behavior.

**Table 15:** The hierarchical regression analysis results of family characteristics of the poor against skeptical strategies.

Model		Non-standardized coefficient		Standard coefficient Beta	*t*	*F*	*R* ^2^	Adjusted R^2^
		*B*	*SE*					
1	Constant	2.785	0.125		22.297***	0.438	0.001	−0.001
	Family size	−0.035	0.052	−0.030	−0.662			
2	Constant	2.835	0.139		20.380***	0.552	0.002	−0.002
	Family size	−0.030	0.053	−0.026	−0.567			
	Poverty duration	−0.026	0.031	−0.037	−0.817			
3	Constant	2.675	0.176		15.214***	1.107	0.007	0.001
	Family size	−0.034	0.053	−0.030	−0.651			
	Poverty duration	−0.018	0.032	−0.026	−0.569			
	Poverty degree	0.070	0.047	0.068	1.488			

***Indicates significance at the 0.001 level.

(3) The gain validity of the social characteristics of people with low incomes on the skeptical strategies. Table 16 presents the results of the hierarchical regression analysis of the gain validity of the skeptical strategies on the social characteristics of the poor (intergenerational poverty, main occupation, source of income). Among them, in the first step of regression analysis, the regression coefficient of the predictor “intergenerational poverty” is not significant (*t* = 1.581, *p* = 0.115), and the determination coefficient of the model is also not significant (*F* = 2.499, *p* = 0.115), this factor cannot effectively explain the dependent variable; in the second step of regression analysis, the regression coefficient of the new predictor “main occupation” is not significant (*t* = −0.531, *p* = 0.596), and the new model The coefficient of determination is also not significant (*F* = 1.389, *p* = 0.250), and the new factor cannot effectively explain the dependent variable; in the third step of regression analysis, the regression coefficient of the new predictor “source of income” is not significant (*t* = −1.740, *p* = 0.083). The coefficient of determination of the new model is not significant (*F* = 1.938, *p* = 0.123). The new factor cannot effectively explain the dependent variable. The social characteristics of the poor cannot effectively explain the skeptical strategies of poverty-alleviation behavior.

**Table 16:** The results of hierarchical regression analysis of social characteristics of the poor against skeptical strategies.

Model		Non-standardized coefficient		Standard coefficient Beta	*t*	*F*	*R* ^2^	Adjusted R^2^
		*B*	*SE*					
1	Constant	2.557	0.100		25.679***	2.499	0.005	0.003
	Intergenerational poverty	0.069	0.044	0.072	1.581			
2	Constant	2.593	0.120		21.546***	1.389	0.006	0.002
	Intergenerational poverty	0.068	0.044	0.071	1.559			
	Main occupation	−0.016	0.031	−0.024	−0.531			
3	Constant	2.684	0.131		20.482***	1.938	0.012	0.006
	Intergenerational poverty	0.069	0.044	0.072	1.583			
	Main occupation	0.004	0.033	0.006	0.121			
	Income sources	−0.068	0.039	−0.084	−1.740			

***Indicates significance at the 0.001 level.

Based on the results of the above analysis, the skeptical poverty-alleviation strategies is mainly related to two individual factors: the health status and educational level of the poor. These two factors can jointly explain 2.3% of the total variation of the skeptical strategies. Those poor people with poor physical health and low education are skeptical about the improvement of their poverty status. However, the family characteristics and social characteristics of the poor cannot explain their skeptical poverty-alleviation strategies.

### 4.5 Predictive role of socio-demographic characteristics on helplessness strategies

(1) The individual characteristics of people with low incomes affect the gain validity of the helplessness strategies. Table 17 presents the results of the hierarchical regression analysis of the individual characteristics of people with low incomes (age, health status, education level) on the gain validity of the helplessness strategies. Among them, in the first step of regression analysis, the regression coefficient of the predictor “age” is not significant (*t* = 0.764, *p* = 0.445), and the determination coefficient of the model is also not significant (*F* = 0.583, *p* = 0.445). This factor cannot effectively explain the dependent variable; in the second step of regression analysis, the regression coefficient of the new predictor “health status” is not significant (*t* = 1.436, *p* = 0.152), and the coefficient of determination of the new model It is also not significant (*F* = 1.324, *p* = 0.267). The new factor cannot effectively explain the dependent variable; in the third step of regression analysis, the regression coefficient of the new predictor “education level” is not significant (*t* = −0.729, *p* = 0.466), and the coefficient of determination of the new model is not significant (*F* = 1.059, *p* = 0.366). The new factor cannot effectively explain the dependent variable. It can be seen that the individual characteristics of the poor cannot effectively explain the helplessness strategies of poverty-alleviation behaviors.

**Table 17:** The results of hierarchical regression analysis of the individual characteristics of the poor against the strategies of helplessness.

Model		Non-standardized coefficient		Standard coefficient Beta	*t*	*F*	*R* ^2^	Adjusted R^2^
		*B*	*SE*					
1	Constant	2.711	0.104		26.046***	0.583	0.001	0.000
	Age	0.028	0.037	0.035	0.764			
2	Constant	2.634	0.117		22.488***	1.324	0.005	0.001
	Age	0.008	0.039	0.010	0.215			
	Health status	0.068	0.048	0.070	1.436			
3	Constant	2.746	0.194		14.167***	1.059	0.007	0.000
	Age	0.000	0.041	0.000	−0.014			
	Health status	0.063	0.048	0.064	1.297			
	Education level	−0.030	0.041	−0.036	−0.729			

***Indicates significance at the 0.001 level.

(2) The gain validity of the helplessness strategies on the family characteristics of people with low incomes. Table 18 presents the results of the hierarchical regression analysis on the gain validity of the helplessness strategies on the family characteristics of the poor (family size, poverty duration, poverty degree).

**Table 18:** The hierarchical regression analysis results of family characteristics of the poor on helplessness strategies.

Model		Non-standardized coefficient		Standard coefficient Beta	*t*	*F*	*R* ^2^	Adjusted R^2^
		*B*	*SE*					
1	Constant	2.974	0.137		21.671***	2.041	0.004	0.002
	Family size	−0.082	0.058	−0.065	−1.429			
2	Constant	3.016	0.153		19.727***	1.214	0.005	0.001
	Family size	−0.078	0.058	−0.062	−1.350			
	Poverty duration	−0.022	0.034	−0.029	−0.625			
3	Constant	3.109	0.194		16.066***	1.015	0.006	0.000
	Family size	−0.076	0.058	−0.060	−1.303			
	Poverty duration	−0.026	0.035	−0.034	−0.743			
	Poverty degree	−0.041	0.052	−0.036	−0.787			

***Indicates significance at the 0.001 level.

Among them, in the first step of regression analysis, the regression coefficient of the predictor “family size” is not significant (*t* = −1.429, *p* = 0.154), and the determination coefficient of the model is also not significant (*F* = 2.041, *p* = 0.154), this factor cannot effectively explain the dependent variable; in the second step of regression analysis, the regression coefficient of the new predictor “years in poverty” is not significant (*t* = 0.625, *p* = 0.532), and the new model The coefficient of determination of is also not significant (*F* = 1.214, *p* = 0.298), and the new factor cannot effectively explain the dependent variable; in the third step of regression analysis, the regression coefficient of the new predictor “poverty degree” is not significant (*t* = −0.787, *p* = 0.432), and the coefficient of determination of the new model is not significant (*F* = 1.015, *p* = 0.386). The new factor cannot effectively explain the dependent variable. It can be seen that the family characteristics of the poor cannot effectively explain their helpless strategies to escape poverty.

(3) The gain validity of the social characteristics of people with low incomes on the helplessness strategies. Table 19 presents the results of the hierarchical regression analysis of the gain validity of the social characteristics of the poor (intergenerational poverty, main occupation, source of income) on the helplessness strategies. Among them, in the first step of regression analysis, the regression coefficient of the predictor “intergenerational poverty” is not significant (*t* = 1.515, *p* = 0.130), and the determination coefficient of the model is also not significant (*F* = 2.296, *p* = 0.130), this factor cannot effectively explain the dependent variable; in the second step of regression analysis, the regression coefficient of the new predictor “main occupation” is not significant (*t* = −1.507, *p* = 0.132), and the new model The coefficient of determination is also not significant (*F* = 2.287, *p* = 0.103), and the new factor cannot effectively explain the dependent variable; in the third step of regression analysis, the regression coefficient of the new predictor “source of income” is not significant (*t* = −0.912, *p* = 0.362). The coefficient of determination of the new model is not significant (*F* = 1.801, *p* = 0.146). The new factor cannot effectively explain the dependent variable. It can be seen that the social characteristics factors of the poor cannot effectively explain the helplessness strategies of poverty-alleviation behavior.

**Table 19:** The results of hierarchical regression analysis of social characteristics of the poor against helplessness strategies.

Model		Non-standardized coefficient		Standard coefficient Beta	*t*	*F*	*R* ^2^	Adjusted R^2^
		*B*	*SE*					
1	Constant	2.629	0.110		23.981***	2.296	0.005	0.003
	Intergenerational overty	0.073	0.048	0.069	1.515			
2	Constant	2.741	0.132		20.729***	2.287	0.009	0.005
	Intergenerational overty	0.070	0.048	0.066	1.460			
	Main occupation	−0.051	0.034	−0.068	−1.507			
3	Constant	2.793	0.144		19.357***	1.801	0.011	0.005
	Intergenerational overty	0.071	0.048	0.067	1.471			
	Main occupation	−0.040	0.037	−0.053	−1.084			
	Income sources	−0.039	0.043	−0.044	−0.912			

***Indicates significance at the 0.001 level.

The above analysis results show that factors such as the individual characteristics, family characteristics and social characteristics of the poor have nothing to do with the formation of helplessness strategies to get rid of poverty, and these factors cannot effectively predict the helplessness strategies of the poor.

## 5 Discussion

This study investigates the predominant models of poverty-alleviation strategies among poor populations in rural areas of China, and examines the predictive role of their socio-demographic characteristics on these strategies. The research finds that the poverty eradication behavior strategies of the poor population are mainly manifested in four forms: fatalism, pragmatism, skepticism, and a sense of helplessness. Those holding a fatalistic strategies typically believe that wealth and poverty are predestined, and that individual efforts cannot alter their poor status. They feel like useless individuals who are difficult to be accepted by society, and hold no hope for the future. This strategies ranks first among all four and can explain 29.14% of the total variance. It is the most common response to poverty among rural poor population, reflecting the mainstream mentality toward poverty-alleviation. It is worthy of high attention from poverty-alleviation workers, who should focus on changing the fatalistic thoughts and behavior patterns of the poor population as a key aspect of psychological poverty-alleviation work. Those with a pragmatic approach usually have full confidence in overcoming family poverty, exhibit an optimistic attitude, believe they can face realistic family problems, respond sensibly to family life difficulties, and are capable of managing family affairs. This is the only positive strategies among the four, accounting for 12.30% of the total variance. Although those holding a pragmatic view do not yet constitute the mainstream force in poverty-alleviation work, they represent a positive and healthy force among the poor and serve as a model in poverty-alleviation practice. Individuals with a skeptical outlook often lack goals for poverty-alleviation, have no expectations for the family’s future, lack improvement plans, and are skeptical about their ability to escape poverty. This strategies accounts for 7.08% of the total variance. Those feeling helpless typically fail to see their own strengths, are powerless to change their situation, feel inferior, and are unable to control their negative emotions. This strategies explains 5.56% of the total variance. Although skepticism and helplessness are not the dominant strategies among the rural poor population, those holding these strategies often become the challenging group in poverty-alleviation work due to their lack of endogenous impetus for poverty-alleviation. The essence of this endogenous impetus lies in the exertion of self-efficacy and the realization of self-worth (Zheng, 2019; Mo and Zhang, 2018), encompassing the desire, confidence, courage, and perseverance required to escape poverty. Endogenous impetus serves as both the ideological impetus and behavioral impetus necessary for achieving sustainable poverty-alleviation goals (Hang and Hu, 2017; Liu and Ma, 2017; Yang and Huang, 2019) and is an indispensable factor for the poor population in overcoming poverty. Empirical research indicates a significant positive correlation between the income of poor families and their level of endogenous impetus (Guan et al., 2019). Xue (2017) categorizes approaches to improve the living conditions of the poor population into two primary types: increasing the accumulation of livelihood capital and fostering endogenous impetus. Liu et al. (2017) further argue that, compared to economic poverty based on livelihood capital, subjective poverty at the individual psychological level can more effectively reflect the poor population’s poverty status. In other words, stimulating endogenous impetus can be more effective than the accumulation of livelihood capital in helping the poor population escape poverty. However, the lack of endogenous impetus among the rural poor population significantly constrains the efficiency of China’s poverty reduction investments and the speed of its poverty-alleviation process (Xu, 2013). Given the various behavioral characteristics exhibited by the rural poor population due to their insufficient endogenous impetus, we believe that stimulating this endogenous impetus should start with enhancing their self-development capabilities. This involves increasing participation in skill training, strengthening their ability to resist risks, and fostering a firm belief in a better life. These measures will encourage the rural poor population to proactively seek pathways out of poverty.

In summary, this study indicates that the combined explanatory power of “Fatalism” and “Pragmatism” poverty-alleviation strategies accounts for over 40% of the total variance, underscoring their critical importance in improving the poor status. This should garner significant attention from poverty-alleviation practitioners. Among these strategies, the “Fatalism” approach is currently prevalent and represents the dominant mentality toward poverty within rural poor population. Given its potential to obstruct the achievement of comprehensive poverty-alleviation goals, it should be a focal point in psychological interventions aimed at poverty-alleviation. Conversely, the “Pragmatism” strategies embodies a healthy and proactive approach to poverty-alleviation. It holds profound and extensive significance for achieving victory in the fight against poverty, advancing deep reforms, ensuring social stability, fostering socioeconomic development, and ultimately realizing a moderately prosperous society. As such, this strategies merits prioritization in both the excavation and cultivation efforts within psychological poverty-alleviation practices. The practice of psychological poverty-alleviation emphasizes the development of a healthy personality and mindset. The internal psychological experiences and motivations of the rural poor population significantly influence their responses to and engagement with external poverty intervention measures, constituting a critical component in improving the efficacy of targeted poverty-alleviation initiatives (Jiang, 2018). Incorporating psychological poverty-alleviation into top-level policy design and establishing a mental health service system for the poor populations are essential steps. Additionally, guiding societal perceptions of poverty accurately and encouraging diverse stakeholders to engage in psychological poverty-alleviation efforts are crucial. Cultivating robust personalities among individuals and nurturing a positive mindset within poor groups are also vital components (Xie, 2019).

This study also found that the socio-demographic attributes of rural poor population exhibit distinct patterns in predicting various poverty-alleviation strategies, with the pragmatic approach demonstrating the broadest predictive efficacy. This strategies is not only associated with individual characteristics such as age, health status, and educational level but also linked to social factors including family size, depth of poverty, main occupation, and income sources. These factors collectively account for approximately 10% of the variance in the pragmatic strategies. Conversely, the fatalistic approach has a narrower predictive scope and weaker predictive power. It is primarily related to individual attributes such as health status and educational level, as well as social features like income sources, with these characteristics jointly explaining less than 5% of the variance. The predictive influence of socio-demographic attributes on the skeptical strategies is even weaker, with only health status and educational level accounting for 2.3% of the total variation in this strategies. The helplessness strategies, however, is entirely unrelated to socio-demographic attributes. Overall, socio-demographic characteristics have relatively weak explanatory power regarding the poverty-alleviation strategies adopted by rural poor population. Nonetheless, compared to other strategies, the pragmatic and fatalistic approaches have a more pronounced sociocultural foundation. Enhancing the socio-demographic attributes of the poor population can, to some extent, facilitate the adoption of a pragmatic poverty-alleviation strategies while mitigating the reliance on fatalistic strategies. For instance, widespread implementation and improvement of the rural poor population medical insurance systems, enhancement of rural basic education and vocational training conditions, expansion of employment and income opportunities for farmers, and control of family size are measures that not only promote economic poverty-alleviation but also improve the attitudes and behaviors of the poor population toward their state of poverty. According to researchers (Abraham and Kumar, 2008), if adults receive an additional 2 years of continuing education, it could result in 60 million people escaping poverty; completing secondary education could lift 420 million people out of poverty, and each additional year of education can increase an individual’s income by 10%. Therefore, education is a crucial pathway out of poverty (Heckman, 2007). Increasing investment in education is fundamental to eradicating poverty and constitutes a key measure in poverty-alleviation efforts (Ushadevi, 2001; Psacharopoulos and Patrinos, 2004). Therefore, increasing educational investment in the poor regions and enhancing the cultural and psychological competencies of the rural poor population establishes a robust foundation for their future development.

## 6 Conclusion

This study addresses the practical issue of rural poverty in China and explores poverty-alleviation behavior strategies among the rural poor population from a psychological perspective, using empirical data analysis. It also examines the predictive role of socio-demographic factors on the psychology and behavior of poverty-alleviation among this group. The research yields the following conclusions: (1) The poverty-alleviation behavior strategies of the rural poor population mainly manifest in four forms: fatalism, pragmatism, skepticism, and helplessness, with fatalism and pragmatism being crucial for poverty-alleviation efforts. Therefore, the focus of rural poverty-alleviation efforts, especially psychological support, should be on actively fostering a pragmatic approach to poverty-alleviation while eliminating the fatalistic approach. (2) socio-demographic characteristics such as health status, educational level, family size, main occupation, and income sources of the rural poor population have certain predictive power regarding the formation of pragmatic and fatalistic poverty-alleviation strategies. Consequently, implementing more extensive and effective rural medical insurance systems, education systems, employment and income distribution systems, and fertility policies can promote the formation of pragmatic poverty-alleviation strategies and eliminate fatalistic ones.

In summary, this study provides a distinctive viewpoint that enhances our comprehension of the essence of poverty, elucidates the structural attributes of behavior strategies employed in poverty eradication, and examines the influence of socio-demographic variables on the anti-poverty actions of rural poor population. The insights gained from these investigations are instrumental in constructing a theoretical and practical policy framework for poverty-alleviation with distinct Chinese characteristics, thereby contributing to the development of an academic discourse on psychological approaches to poverty-alleviation imbued with Chinese elements. Moreover, these findings play a pivotal role in devising and implementing enduring mechanisms for poverty-alleviation policies. They offer valuable guidance for the pursuit of sustainable and effective poverty-alleviation strategies, which is essential for advancing the global endeavor against poverty.

## Data Availability

The original contributions presented in the study are included in the article/supplementary material, further inquiries can be directed to the corresponding author.
